# Analysis on Use of Open Educational Resources during EmergencyRemote Education due to Covid-19: A Case Study in Turkey

**DOI:** 10.1177/21582440221130299

**Published:** 2022-11-19

**Authors:** Ayşe Saliha Sunar, Esra Yükseltürk, İsmail Duru

**Affiliations:** 1 Bitlis Eren University, Bitlis, Turkey; 2 OSTIM Technical University, Ankara, Turkey; 3 Istanbul Sabahattin Zaim University, Istanbul, Turkey

**Keywords:** open educational resources, emergency remote teaching, distance learning, Covid-19, higher education, change in educational practices

## Abstract

During the mass curfews, travel bans, school shutdowns, face-to-face education was discontinued, and many universities had to urgently switch to online education. Academics, most of whom are not familiar with digital pedagogy, had to adapt their lectures to online learning. The aim of this study is to analyze how Open Educational Resources (OERs) are used in practice during emergency remote teaching (ERT) and whether this influences the regular practice of academics on a global level and in Turkey in the longer term. Methodologically, we adopt a mixed-methods approach in two stages: (i) an empirical study conducted in Turkey to find out what prior knowledge and experience academics have with OERs and how they use OERs during ERT; (ii) a complimentary desk study on the global situation of OER use. Our results show that academics who did not know about OERs before the pandemic are still hesitant to use them, even though they have prior experience with online teaching. In addition, academics with higher rank and academics in architecture, philology, and arts have the most negative opinion about campus education being fully accessible online.

## Introduction

The novel Coronavirus disease, Covid-19, first appeared in Wuhan, China, in late December 2019 (Rothan & Byrareddy, 2020). The Covid-19 virus has spread the whole world unexpectedly in a short time. On 23 January 2020, the first curfew was imposed in Wuhan to reduce the risk of further transmission of the disease (World Health Organization [WHO], 2020).

With the tightening of curfews around the world, the situation of educational disruption has arisen. It has led to the widespread closure of schools, colleges, universities, and other educational establishments in many countries in order to stem the spread of Covid-19. As a result, the face-to-face (F2F) campus education offered by higher education institutions was stopped to adapt to the new situation and required an emergency transition to online education.

The institutional basis of distance learning goes back to the time long before the invention of the Internet and the World Wide Web (WWW) (Rumble, 1989). The evolutionary speed of distance learning, however, increased with the invention of the Web. Educationalists discussed that the evolution of the Web from Web 1.0 to Web 2.0 would inevitably impact education by providing teachers and learners with a variety of online tools, what is known as Education 2.0, in the early 2010s. Nevertheless, the evolution of the Web has definitely had an impact on the way universities work in particular. For example, the promotion of open educational resources (OERs) by UNESCO has influenced the way universities make their curricula and lecture notes publicly available.

OERs are defined by UNESCO as educational materials that are publicly available or licensed by an open license allowing free access, use, adaptation, and redistribution by others with no or minimum restrictions (UNESCO, 2011). The motivation behind the participation and endorsement of OERs by organizations such as UNESCO is to provide high-quality education and educational content at low cost to more people, including those who cannot afford it worldwide (Hylén, 2006).

Given the low-cost (free of charge, limited access at no cost, or full access at a discounted rate) online access to OERs, they could be a great resource for use in emergency online education, where everything from lecture notes to classrooms is web-based (Reimers et al., 2020). However, their use requires prior knowledge and experience for both academics and students. This paper aims to analyze (i) how academics from Turkey have used OERs during the rapid transition to online education and (ii) whether their attitudes changed toward OERs and online education. In addition, mainly to show the differences between countries, an investigation was conducted on how the world uses OERs during emergency online education.

## Related Work: OERs and Emergency Remote Teaching

A reliable and strong distance education system requires an investment of time and money to train teachers and students, provide the necessary infrastructure and tools and provide a solid curriculum for the class. The rapid transition to remote online education forced by Covid-19 cannot be considered a normal application of online teaching. It is called *online emergency remote teaching* and certainly has shortcomings (Hodges et al., 2020).

The rapid development of distance learning and the growing number of web-based applications for education has given rise to some unrealistic claims from the media and academia. For example, it is claimed that Massive Open Online Courses (MOOCs) as an open and distance education application were to bring down on-campus education when the first MOOCs started broadcasting (Lewin, 2012). The MOOCs are an implementation of the provision of OERs and offer structured courses that are freely available to lifelong learners and can also be used in blended campus education. However, the way universities have dealt with emergency remote teaching due to Covid-19 confirms once again that despite the hopeful projections for the future, we are far from the massively adopted distance education into higher education (e.g., Kollalpitiya et al., 2020).

According to UNESCO, more than 190 countries have closed schools and educational institutions, affecting almost 90% of the world’s student population as of 13 May 2020 (UNESCO, 2020). The very challenging spring semester of 2020 has shown that, in most cases, universities are not yet ready for change on this scale, even though they have distance learning centers (Crawford et al., 2020; Reich et al., 2020). There are studies that examine the measures implemented by universities in order to continue education (Bozkurt et al., 2020; Parthasarathy & Murugesan, 2020).

A 2017 report by EDUCAUSE reported that 91% of academics would not prefer online learning Pomerantz and Brooks (2017). Despite the preferences of academics, the global pandemic most likely forced academics to prepare online lectures in a very short time without knowing the basic principles of online learning Haugsbakken et al. (2019). Academics had to adapt their courses, which are most likely prepared for face-to-face teaching, into online education, which is a very new concept for many academics. During the emergency transition to remote education, the institutes offered training for the teachers, but the main focus mostly was on the use of e-learning tools. Very few of them offered a training course on creating online content accessible to the vast majority of learners, maintaining student motivation, and how to evaluate learners online (Nabukeera, 2020; Watermeyer et al., 2021). During this rapid transition, OERs could be a great resource for academics to prepare their online teaching materials. However, the lecturers may not know how to reach them or how to integrate them into their courses, as this is an issue that educational technologists are concerned with even before the outbreak (Chotto & Rivera, 2017; Ganapathy et al., 2015; Sunar et al., 2020).

Zooming into the perception of academics from Turkey, the literature provides some critical situations. A number of studies reported how academics in Turkey perceived distance learning, open educational resources, and MOOCs. The studies conclude some common findings as follows:

- The majority of academics are not willing to give lectures online because they believe face-to-face education is one of the key factors for success (Koloğlu et al., 2016).
- Academics usually believe that online education alone is not enough; it should be blended or used only for lifelong learning, not for a degree (Gürer et al., 2016).
- If academics find it easy to use the technology, they are more willing to change their traditional tool and blend them with technology, which has a positive correlation with the success of using online education (Turan & Çolakoğlu, 2011).

This being said, we should mention the theory that explains the stages of adoption of an innovation. Diffusion of innovation refers to the process that occurs when people adopt an innovation such as a new idea, a new product, a new practice, and a new philosophy (Kaminski, 2011). The theory explains the stages, including the change of mind and behavior in accepting and practicing the innovation. According to this theory, there are five stages in the adoption process of innovation: Knowledge or Awareness Stage, Persuasion or Interest Stage, Decision or Evaluation Stage, Implementation or Trial Stage, and Confirmation or Adoption Stage. In this work, academics were asked various questions to understand whether they are aware of the innovation, that is, OER and online education, whether they are changing their educational practice, and whether they aspire to maintain this change in educational practice in the future. According to this theory, the reasons for academics’ tendency not to use online lectures and thus to use OERs could be at different levels and caused by so many different reasons and experiences. Therefore, we aim to examine the readiness of academics in terms of pedagogical and technological information and applications. The term “readiness” refers to any level of the adoption process from awareness to the use of digital pedagogy, digital technology and tools.

There is, of course, research on OERs available in the literature, such as Otto et al. (2021)’s study investigating trends and gaps in empirical research on open educational resources covering the years between 2015 and 2019. The study shows that most of the empirical research on OERs is about the use and adoption of OERs in higher education, mostly through use cases. Also, the majority of these studies are located in the US—mostly North America. It is also indicated that very little of the published research is specifically about open science and open research. There is a gap in the literature on how academics are adopting OER and related technologies such as open licensing and distance learning as technological innovation, what stage they are at in adopting the innovation, what their experience and background knowledge are on the subject, particularly in the countries other than the US. Our research aims to understand what academics in Turkey, who are at different academic ranks, in different subjects, of different ages and genders, know about OERs and open licenses, how they use these technologies, whether emergency distance education brings about changes in their understanding and adoption of these technologies, and whether they are willing to use them in the long term. Therefore, we investigate the aspects regarding the readiness of academics in distance education and OER materials at knowledge or practice levels. In conclusion, our study provides an analysis of existing practice and the knowledge and experience of academics in emergency remote teaching, particularly with regard to open educational resources.

## Objective and Research Questions

Taking into account all potential difficulties and emergency solutions, the main objective of this study is to examine how willing university lecturers are to teach online, how they are aware of OERs and open licenses, whether they have already used OERs, and how they have used OERs during the recent emergency transition to online learning due to Covid-19. In this study, we seek to investigate answers to these research questions:

- RQ1: What is the example of providing opportunities for a smooth transition to online education in higher education due to the pandemic worldwide, that is, closure of campuses, online lectures, available resources for online education, measures taken for quality online education?
- RQ2: What is the readiness of academics in Turkey in terms of pedagogical and technological information and tools at the knowledge and practice level, that is, knowing, accepting, or using the information and/or tools on digital pedagogy and technology?
- RQ3: Are academics in Turkey aware of OERs and Creative Common (CC) licenses? If so, to what extent have they used OERs in their lecture preparation and made their own resources publicly available?
- RQ4: Is there a change in the habit/awareness of academics in Turkey regarding the use of OERs before and during the pandemic?
- RQ5: If there is a change in the educational practices of academics in Turkey regarding the use of OER before and during the pandemic, is there a difference among academics according to age, gender, the field of study and academic rank?

## Methodology

This study follows a mixed (qualitative and quantitative) methodological approach by conducting an empirical research study with complimentary desk research for a systematic mapping. We conducted an online survey with over 130 academics from Turkey to understand how they used open educational resources in preparing their lectures and the challenges and benefits of an emergency transition to online education for them comparing their readiness before the pandemic. Regarding the collected samples, it provides a limited representation of the academics in Turkey. In order to generalize the findings across the country, bigger research needs to be conducted with a larger proportion of academics. The results analyzed in the related section represent the behavior in the sample case.

The survey was produced in Turkish and distributed via an online tool, Google Forms. The survey consisted of eight parts: general information, transition during the pandemics, readiness for online education, use of OERs, assessment and evaluation, infrastructure and tools, sharing of educational materials open, and post pandemics. Most of the questions are multiple-choice questions. Some open-ended questions to identify their challenges, rationale, and thoughts are added to the end of the parts.

The survey is distributed over online academic network platforms such as LinkedIn and online academic groups. Although we conducted the survey during such a disastrous time when there were overwhelmingly many requests for participation in scientific questionnaires regarding the pandemics in the mailboxes, we reached 133 academics who voluntarily participated in our questionnaire. Our dataset is almost evenly distributed in terms of gender and role of the participants in order to provide meaningful insight into the situation in Turkey. The results are presented in Section *Emergency Remote Teaching: A Case Study on Higher Education in Turkey.* To provide complementary results for the empirical study on Turkey, desktop research on the worldwide use of OERs during emergency remote education is also conducted by using (i) academic articles on academic prospectus databases, that is, Web of Science and Google Scholar and (ii) online news and reports via the Google Search browser.

## Investigation on Exploitation of OERs during Emergency Transition to Online Education

The Turkey specific analysis is a piece of proof that academics’ awareness and readiness of OERs are satisfactory to thoroughly exploit all the free open resources along with those paid options that are made free during the pandemic. However, Turkey is not a rare example of this matter. Our motivation to prepare this section is twofold: to show (i) how OERs are used as one of the first solutions to ease the process of online education during the global pandemic and (ii) the gap between countries’ use of OERs during emergency online education considering the countries’ infrastructural readiness and wealth. We aim to put a picture of how institutions from different countries around the world have become accustomed to emergency remote teaching using OERs to support the educational process.

Some universities have already established infrastructure for distance education and have already practiced blended teaching on campus long before the pandemic. For example, Harvard University has continued to use the existing distance learning tools that were already in use before the pandemic. The university also developed a platform to collect resources on best online practices, tools, and support for online learning. For synchronous online lectures, Harvard University preferred the Canvas platform as a learning management system that integrated a web conferencing system, Zoom.

As an example, apart from the Ivy League universities, the University Utara Malaysiahas already introduced blended learning in recent years, where students learn online as well as F2F. Therefore, the transition to online learning was very smooth during the Covid-19 pandemic.

Singapore, like Malaysia, is one of the countries with the least difficulty in the process, thanks to its technological infrastructure. Singapore-based technology company Larkannounced that due to Covid-19, it has been making its digital collaboration tools available free of charge in the Southeast Asian region, including Singapore, Malaysia, Indonesia, the Philippines, Thailand, and Vietnam, since March 2020. UNESCO has also recognized all of this along with others from the world as a platform that can make learning easier for students, parents, and teachers and enable social interaction during Covid-19.

Higher education institutes exploit all the freely and openly tools available to ease the transition. OER platforms, including MOOCs, therefore, are one of the immediately available resources. For instance, the Italian MOOC platform Federica.eu, which supports over a hundred University Naples Federico II (UNINA) professors, uses Federica MOOCs and uses Microsoft teams to deliver online lessons (Kerr, 2020). UNINA transferred 3,500 courses, 3,200 exams, and 270 final degrees to the online space.

Slovenia’s VideoLectures.Netthe UNESCO award-winning freely accessible repository for educational video lectures represents a nice example of how OERs are transformed during the pandemic. VideoLectures.Net offered conference organizers the service of holding the conferences online and publishing the content free of charge, as many conferences have to be held online due to the pandemic travel restrictions.

**Figure 1.**
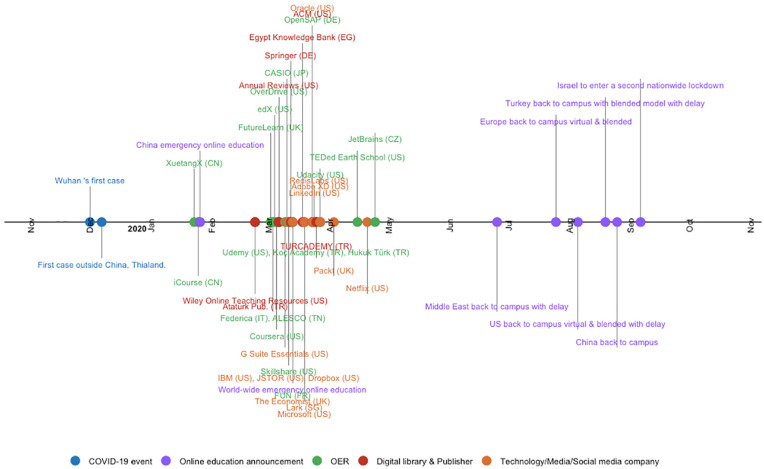
Private companies and open education initiatives showed immediate support by making their content freely available to support students and instructors.

The private companies also contributed to the support for distance education. For example, Telecom Egypt provides free access to the Egyptian Knowledge Bank, which was identified as a fairly reliable source that was used during the transition (Shehata et al., 2020). More examples of companies from around globe are shown in Figure 1.

To show the collaborative effort at the global level for supporting education during the pandemic, we created a timeline in Figure 1. The figure shows some already freely accessible sources together with paid resources that are offered for free by companies during the lockdowns. For clarity, we have selected examples from the available samples to cover different countries, even if the for-profit companies were predominantly based in the United States.

These examples can be considered as a representative that digital technologies in the form of open content, open licenses, open formats, and open software have the potential to break down some of the barriers in education (Lane, 2009). However, Lane (2009) analyses excluded communities and divisions in reaching the OERs. The author shows that there is a gap in reaching digital technologies and OERs that open further opportunities for open and online learning among groups of people in societies that depend on the infrastructure of technology in their area, the availability of tools to reach the OERs, digital literacy, digital competence, and so on.

The transition to emergency remote teaching in some regions shows that the transition has not always been smooth and competent. Universities with inadequate technological infrastructure are most affected by this process. For example, Universidad Mayor de San Andrés (Bolivia) has suggested that educators use generic applications such as email, video calls, and WhatsApp in the absence of a virtual campus.

There are attempts to overcome the regional handicaps with the aid of OERS. For example, Sub-Saharan Africa is already disadvantaged with its infrastructure, that is, low internet density, low electricity and mobile phone access, which are essential for online learning (Azolibe & Okonkwo, 2020). OER Africa shared resources to support further learning. Kwame Nkrumah University of Science and Technology (KNUST)OER, FundaOER, and OpenUCT, institutional open access repository of the University of Cape Town (UCT) are some examples of OER platforms for the African context.

In addition, the countries already struggling with wars and poverty have difficulties in taking measures against the pandemic. For example, according to UNESCO, Palestine suffers from obstacles to e-learning, mainly infrastructure, weak internet networks, power cuts (especially in Gaza), and a lack of awareness among students and their families of the importance of e-learning. Keeping in mind these obstacles, Palestinian universities also applied online distance learning through video conferencing tools and free social media platforms such as YouTube.

## Emergency Remote Teaching: A Case Study on Higher Education in Turkey

Universities in Turkey rapidly switched to online education in March 2020 and continued online in the Spring 2021 semester. This section provides analyses of the survey conducted with academics from Turkey on how OERs are used during emergency remote teaching. According to the Higher Education Council of Turkey (YOK), 123 out of over 200 universities that have Distance Education Application and Research Center (UZEM), over 90% have made an urgent switch to online education in March 2020. However, not all of them have an adequate infrastructure to offer their students decent distance learning. During the pandemic, new centers for distance education opened. However, there is a lack of evidence to say they are fully operational. In addition, the YOK Open Courseware project promotes the uploading of lecture notes to the platform - initially, four universities are contributing to the collection. Currently, lecture notes from 27 bachelor and 47 pre-bachelor courses, 2,408 books for 35 departments of eight faculties and 2,320 books for 46 pre-bachelor courses are freely available. However, almost all openly accessible materials are written notes and books, which shows that higher education institutions in Turkey do not have properly prepared lecture notes for interactive online education (Ertuğ, 2020).

Apart from the state resources and the international companies, many universities have already published MOOCs in Turkish such as Akadema, Atademix, and Turkcell Academy. In addition to these platforms, Khan Academysupports the Turkish language, and Koc University is a partner of Coursera. However, the amount of content is still very limited; in turn, the participation level remains low (Yürük et al., 2020). The accessibility of the Turkish MOOC platforms is also unsatisfactory, that is, there is a lack of audio and text support for disabled students (Akgül, 2018). The availability of open and accessible resources also shows that universities in Turkey are not ready for a change in educational practice on this scale.

The pandemic process has also shown that both students (Saritaş & Barutçu, 2020) and educators (Sayan, 2020) have not completely possessed the digital competencies and skills needed (Sezgin & Fırat, 2020). At the same time, content, material development and measurement and evaluation issues have been important problems of distance education in this process (Ozer & Suna, 2020).

The remainder of the section analyses the survey responses of 134 academics from Turkey on how they use the OERs, global and local ones, in emergency remote teaching.

### General Description on Data

More than 130 academics who are officially employed at a private or state university in Turkey participated in the survey. Of all participants, 86% work for a state university. The participants who work for a state university are evenly distributed: half of them work for a newly founded university, while the other half are older than 13 years. It should be noted that with the project “one university in every city” launched by the Turkish government, many small universities have been established since 2008 (Özoǧlu et al., 2016). We think that this is an important factor to be expressed as the newly founded universities may not be well equipped. Therefore, it could influence the interpretation of the results.

**Figure 2.**
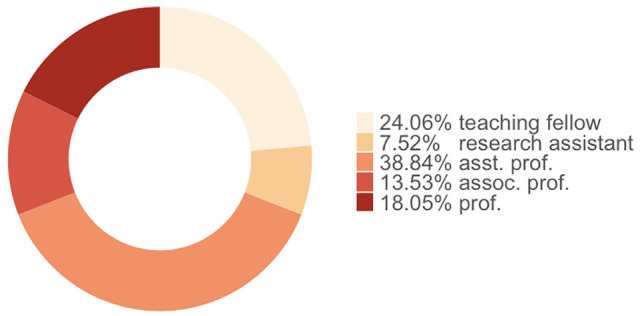
Academic roles of the participants at their universities.

**Figure 3.**
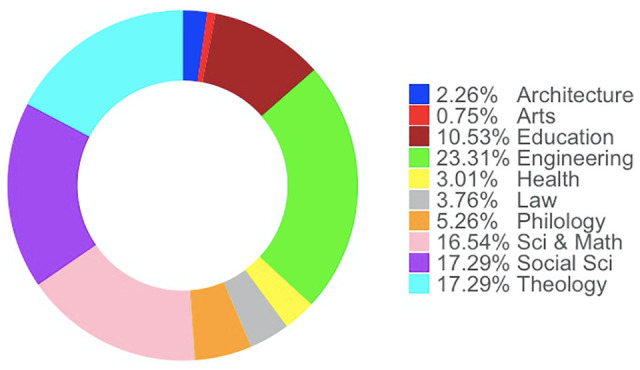
Faculty distribution of the participants.

Figure 2 shows the academic role of the survey participants, and Figure 3 shows the faculty distribution of the participants. The participants come from different faculties, from social sciences to engineering and health. The role of academics is also smoothly distributed. It should be noted that research assistants who have a PhD can give a lecture, while those who do not have a PhD unofficially help the principal lecturer of the class in laboratory classes. Therefore, they are the least populated group in our data. The diversity by gender has also remained fairly constant in the collected data, with 54% of the participants being male while 46% are female.

**Figure 4.**
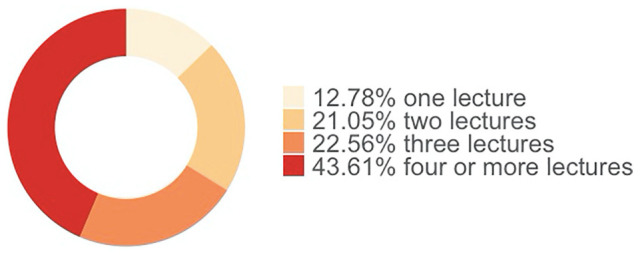
Number of classes given by an academic during emergency remote teaching.

**Figure 5.**
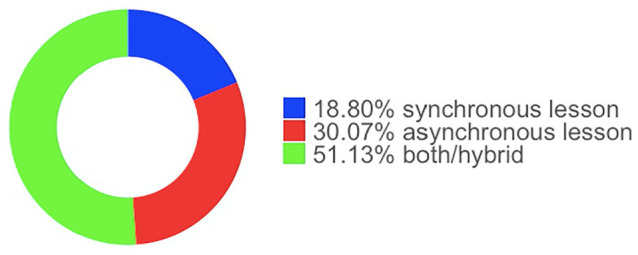
Type of classes given by an academic during emergency remote teaching.

Figure 4 shows that almost 70% of the participants give three or more lectures per week in remote education. While more than half of them hold their lectures synchronously (i.e., through tools such as Zoom and Microsoft Team), 30% of them hold their lectures both synchronously and asynchronously (see Figure 5).

Participants were also asked whether they thought their lectures were suitable for remote education: 44% of them replied as partial, 37% of them said yes, only 19% of them said it was not a suitable instrument for the content of their lecture (e.g., a laboratory class). This result could be interpreted as an encouraging result that the lecturers could be motivated to keep their lectures online with constructive help.

### Academics’ Readiness on OERs and Distance Education

Our results show that the majority of participants (78%) are neither experts in distance learning nor have they been trained in the design of a distance learning course. Moreover, 70% of them have never had any experience in delivering a lecture online.

**Figure 6.**
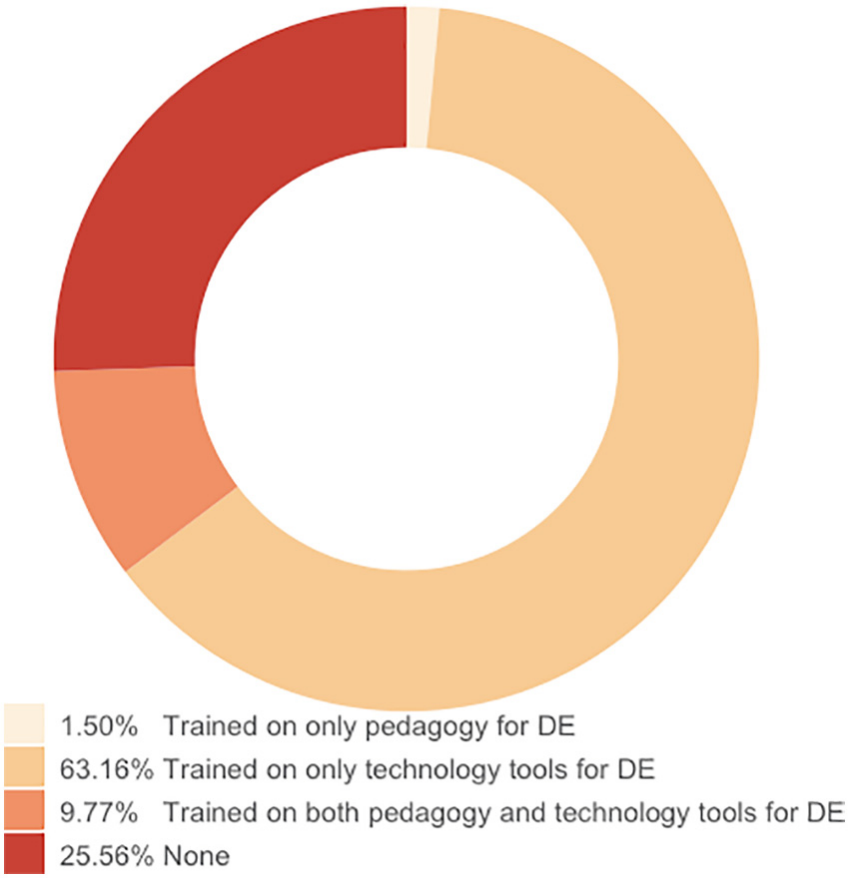
Received training courses on pedagogy and technology for distance education during emergency remote teaching due to COVID-19.

Based on these findings, it is becoming increasingly important to provide lecturers with the necessary training on designing, delivering, and evaluating an online course. Figure 6 shows that 25% of them have never had any training on how to design and deliver a distance learning course or what tools can be used for distance learning during the period of urgent transition to remote education. The majority of respondents (63%) are trained in how to use certain technological tools for remote education. Only 9.8% of them stated that they are trained in both pedagogy and technological tools for remote education.

In addition, 62% of respondents said they had some difficulties in digitizing their lecture notes for remote education. The main challenges are identified as follows:

- Limited time for the digitization of all lecture notes (44%)
- Effective use of the time allocated to the online lecture and appropriate preparation of the materials (23%)
- Prepare interactive slides (12%)
- Search for the right material/tool for the presentation of mathematical equations, laboratory equipment etc. (12%)

### Academics’ Reflection on OERs and Distance Education

The survey seeks to understand whether academics are aware of the OERs and include them in their course preparation in addition to intellectual property licenses.

**Figure 7.**
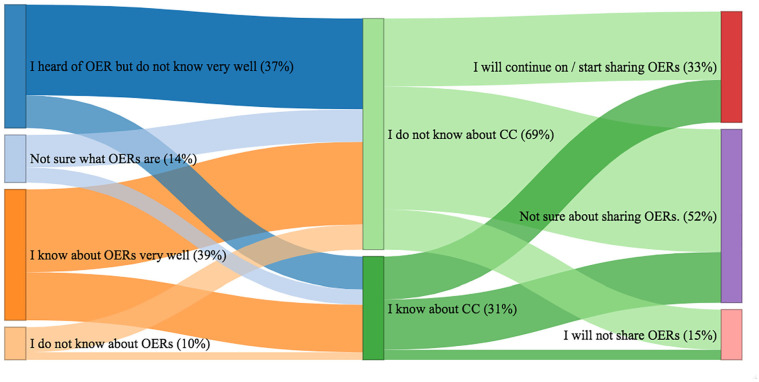
Academics’ readiness and perspectives to benefit from the OERs and share their OER materials.

The Sankey diagram in Figure 7 is to show the proportion and transition between groups of respondents regarding their experiences with OERs and CC licensing. While 39% of respondents said they know about OERs well, 37% said that they have heard about it but do not know it well. Almost 10% of them said they did not know about it and 14% of respondents said they were not sure what an OER is.

This result is consistent with another finding from the survey, which found that over 40% of them never used openly and freely shared educational resources in their course preparation. The most frequently used OER resources are: (i) social media such as YouTube and personal blogs (32.3%), (ii) open coursewares of national universities (31.6%), and finally, (iii) a national platform for university courses from the Higher Education Council of Turkey(18.8%). One of the most common concerns expressed in the open questions of the survey is that it is difficult to find OERs authored in Turkish. This result could explain why the three commonly-used repositories are from national/Turkish sources.

A noteworthy result is that some of the respondents who said they had never heard of OERs also indicated that they benefit from resources such as open national courseware. This result shows that some academics use OERs without knowing the theoretical concept of OERs. Considering the theory for adoption of an innovation, some academics are not even at the first stage, Knowledge or Awareness.

When it comes to MOOCs as a specific OER, most academics in Turkey have never heard of them even before the pandemic (43%). Only 22.6% of respondents said that they know MOOCs well. Not surprisingly, only 9.8% of academics use a MOOC in their class, either fully or partially. The studies show that experts in distance learning consider MOOCs to be a promising development that we should incorporate into the Turkish education system (Kesim & Altınpulluk, 2014). However, students (Kurt, 2019) and academics who are not experts in distance learning (Koloğlu et al., 2016) are usually quite suspicious and do not believe that distance learning is a useful method at all.

The respondents were also asked whether they had ever heard of the CC License. Although there are other open licenses, CC is one of the most widely known, which is why it is explicitly mentioned in the survey. CC License is a standardized way of granting copyright permission for creative works that creates a balance within the traditional “all rights reserved” setting created by copyright law.

Almost 70% of respondents said that they did not know what CC is. In addition, 29% of respondents are not aware of the need to share copyright licenses when making their own materials available in a non-public area. Figure 7 shows the proportion of academics by their attitude toward open sharing of their own materials. The majority of academics who are unaware of the CC license are also unsure about sharing or have chosen not to share. While almost half of the academics who know about CC are also not sure about sharing OERs, very few of them indicated that they would certainly not share OERs. In addition, the answers to the open-ended questions indicate that one of the concerns is who the owner of the intellectual property of the materials created for the course is. Apart from this, some academics are reluctant to openly share their own materials with the public because they do not want to reveal their intellectual property. In addition, some of them are concerned about criticism from their colleagues and managers of the content and quality of their work.

There are also many misconceptions and hesitations that academics have when it comes to sharing materials for teaching, which is summarized in Table 1. The results in the table may help to understand why academics resist changes in pedagogical practice, such as the use of OERs in their lecture preparation.

**Table 1.** Hesitation and misunderstanding by academics in the dissemination and use of OERs.

Representative comments from respondents	Additional information on misconception
• I feel like I am stealing someone’s property. • I prepare the materials by mixing other OERs, which in my opinion, is not ethical. • I use figures that are subject to intellectual property rights.	The materials are licensed under copyright licenses (i.e., CC BY) that allow anyone to participate in the 5Rs activities—retain, reuse, revise, remix, and redistribute (Wiley & Hilton, 2018). In most cases, OERs are licensed so that the resource can be used for non-commercial purposes.
• Only the class-takers would be interested. • It is not fair to open the OERs for those who are not included in the program. • I think it belongs to the university.	The owner of the intellectual property of the materials created for the course varies depending on the university’s legislation. With the OER movement, many universities promote the open share of resources with CC licenses, even though they own the resources (Garon, 2018).
• I am afraid of criticism because I am a junior lecturer. • I am afraid that something I have said could be used against me because I censor what I think and say. • I worry about my own intellectual property rights.	UNESCO’s A Basic Guide to Open Education Resources addresses these concerns.
• If I were to openly share lecture notes, it would be like an open university program(Açık Öğretim Fakültesi in Turkish). • F2F lectures, supervision, and tutorial classes are the main differences between the effectiveness of F2F and the Open University program.	It is thoroughly discussed by educationalists that blended learning can be a model that offers distance lectures and F2F training for tutorial classes and supervision.

### Academics’ Future Plan on OERs and Online Learning

What academics think about continuing online education even after the pandemic is over is analyzed in the section. In this section, we present a number of charts to present the opinion of academics by gender, the field of study, and academic role (rank) on whether or not to maintain the change in educational practices forced by Covid-19.

**Figure 8.**
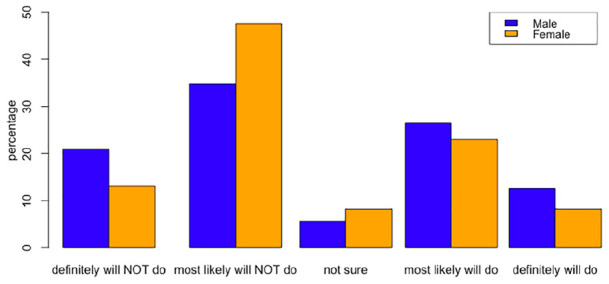
Plan to continue remote education after the end of the pandemic (academics by gender).

The bar graph in Figure 8 shows the opinions of academics by gender on how to continue online education after the pandemic. According to the graph, although the general trend in preferences is very similar, female academics are more likely not to pursue distance learning.

**Figure 9.**
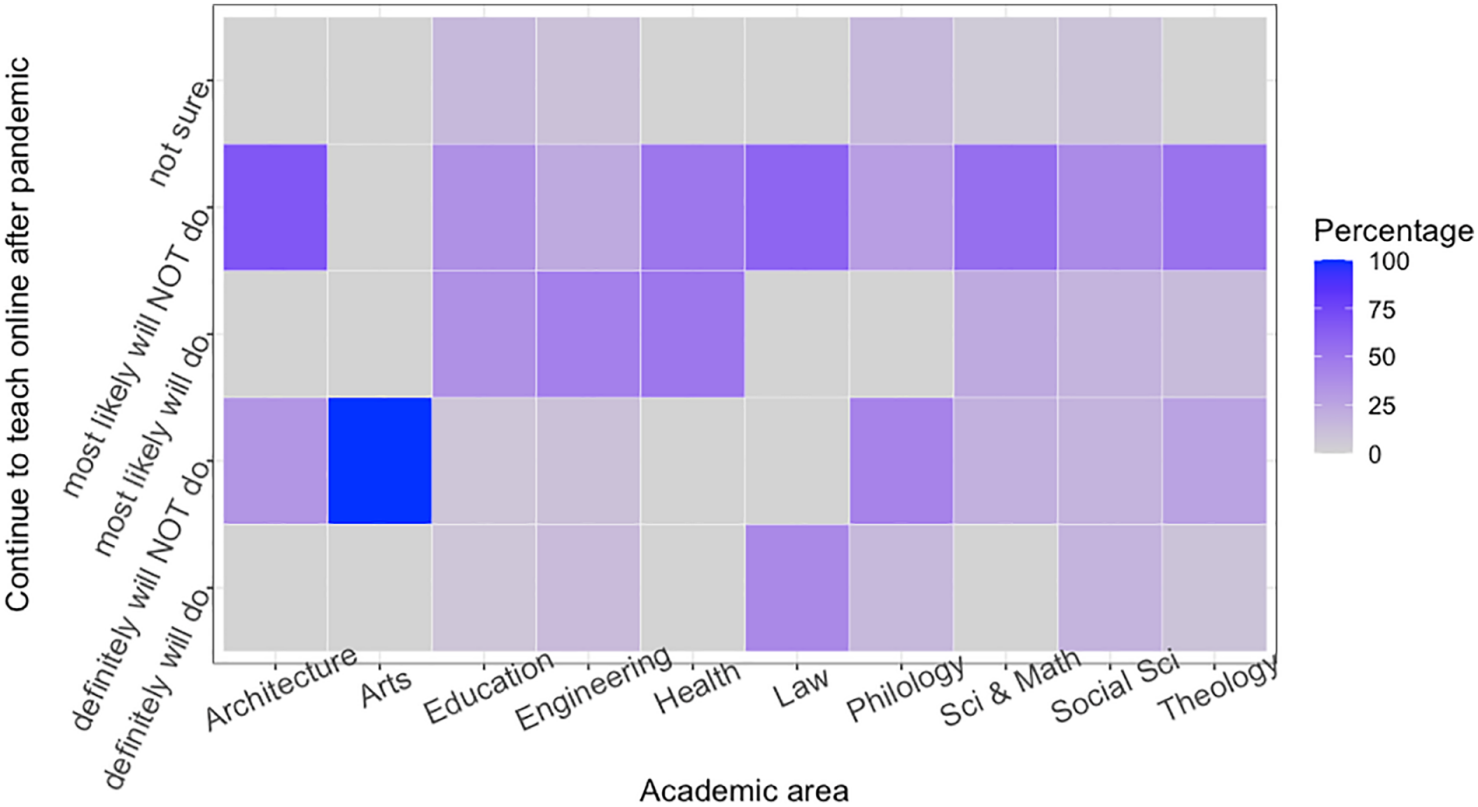
Plan to continue remote education after the end of the pandemic (academics by departments).

The heat map in Figure 9 shows the opinions of academics by the department on how to continue online education after the pandemic.

Overall, the majority of participants, regardless of their expertise, indicated that they would most likely not continue to teach online. In one of the most common answers from academics, it can be observed that they consider open and distance learning to be less valuable. When they are asked about their motivation for their answers, it can be observed that many of them are in favor of blended learning. Among the others, academics in architecture, philology and art have the most negative opinion about campus education being fully accessible online. However, there are also academics from other disciplines who are thinking positively about online education in the wake of a pandemic, such as law, engineering, health and education. It should be noted here that the subsets of groups, that is, academics grouped by the area, are rather small, sometimes less than people. Even though this result implies the overall tendency of the sample case, one should be cautious about generalizing the results to the country or global level.

**Figure 10.**
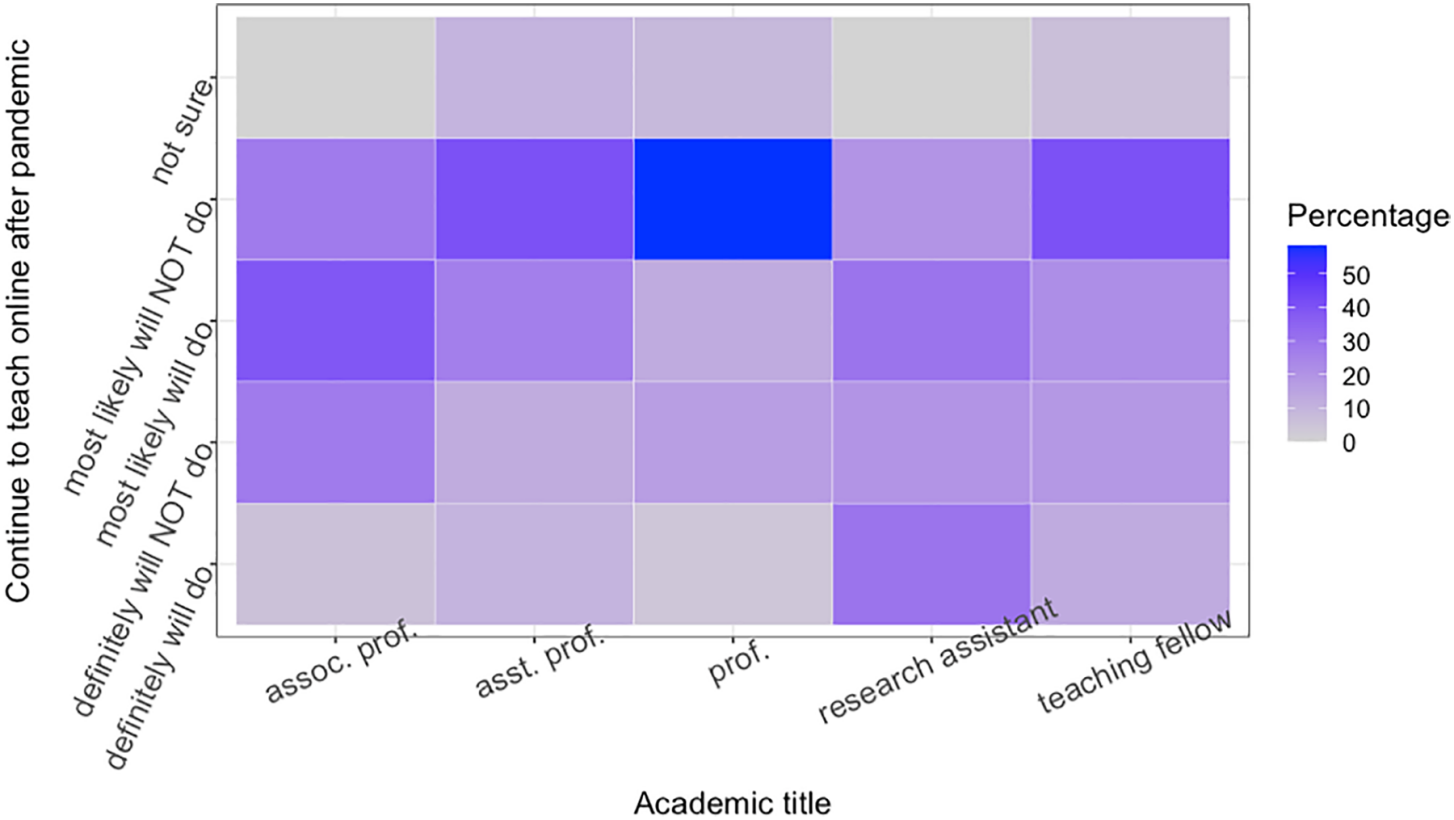
Plan to continue on distance education after the pandemic is over (academics by academic roles).

The heat map in Figure 10 shows the opinions of academics regarding academic roles in online teaching after the pandemic. It can be observed that the majority of professors do not plan to continue online teaching. Associate professors and assistant professors have the most widespread opinions on a scale from “definitely will not do” to “most likely will do.” After the pandemic, the group that is most in favor of online education are research assistants, usually graduate students or newly acquired Master’s or PhD degrees, who are most in favor of online education after the pandemic. Research assistants are generally under 35 years of age (The Turkish Council of Higher Education had an age limit (35 years) regulation for applying for the position of a research assistant. The Council of State abolished this regulation in accordance with Resolution Danistay 8.D. E. 2015/14240, K. 2019/6854, T. 11/07/2019.) It can therefore be interpreted that the youngest group of academics is the most supportive of online education in the future, stating that they “will definitely do” and “most likely will do.” This shows a change in the acceptance and adoption of an (educational) innovation; however, there is a need for further research as to why the youngest group of academics are most positive about online learning.

An interesting result is that the lecturers responsible for the theoretical lessons and tutorials (According to the academic system in Turkey, the main task of lecturers is to give lectures, they are not obliged to do research.) are the second group of academics who usually say that they ’most likely will not do’.

According to the answers to the open questions, most academics stated that blended learning could be an effective way to improve on-campus education.

In order to have a deeper insight into why the academics are mostly hesitant to conduct online teaching, Table 2 summarizes the main reasons why academics are motivated or reluctant to continue teaching online. It has been observed that some lecturers have a misconception about remote education because they believe it is only for emergencies. While some academics are skeptical, others have expressed that they have encountered and effectively benefited from online teaching due to the pandemic. We created the word clouds as a representation of the respondents’ comments, explaining the reasons for their decision.

**Table 2.** The Motivation of Academics to Continue or Not Continue Online Education After the COVID-19 Pandemic.

Motivation to continue online learning
• Flexibility of time and place for students
• Possibility to reach a large number of students
• Class continuity when I travel
• Building and online resources/Archive of my lectures
• Less space required on campus in the computer-based lectures
• Advantage of repeating the lecture for students
• More effective in large classes
• Less bullying in the workplace
• Ambitious with regard to the “schoolless” society
Reluctance to continue online learning
• Different degrees of readiness of the enrolled students
• Cheating in online exams
• Less interactivity and timely feedback from students
• High dropout rate in the class
• Difficulties with the implementation of tutorial lectures
• Immediate intervention in the lectures, if necessary
• Inequality of adequate infrastructure
• Managers underestimate the effort put in online education
• Lack of self-discipline of students
• Problems related to accessibility for disabled students

**Figure 11.**
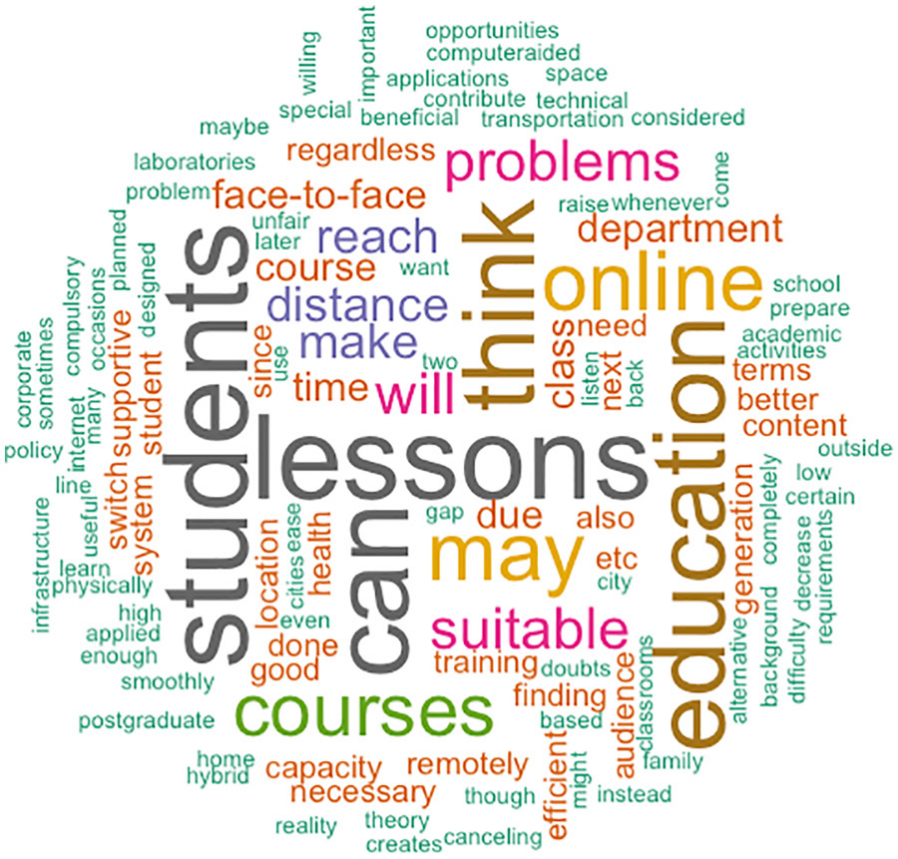
Word cloud of academics who have positive thoughts about continuing online learning after COVID-19.

**Figure 12.**
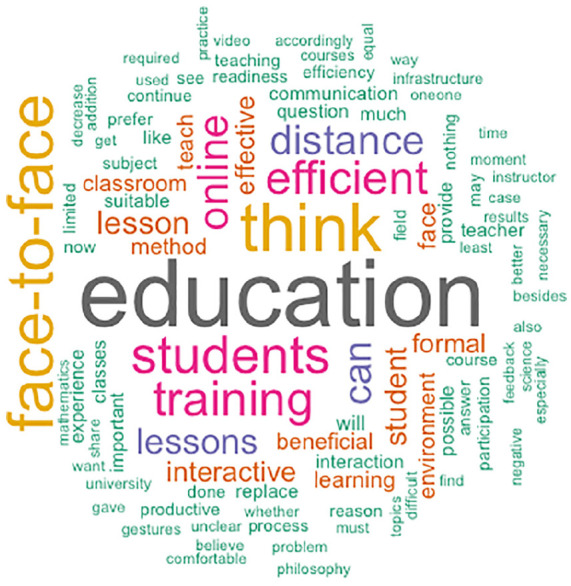
Word cloud of academics who have negative thoughts about continuing online learning after COVID-19.

In addition, to reflect the general perspective of the academics based on their tendency to continue online learning after COVID-19, word clouds are created. These are generated using all the answers given to the open questions in the questionnaire. Some of the words in the word cloud have become notions that have a permanent presence in our life as a result of the pandemic. The phrases originally reserved for people interested in online education have evolved into a notion that is now understood by the majority. Figures 11 and 12 give a general idea of what academics are focusing on as they make further decisions about online education after Covid-19. While words like *education*, *students*, and *distance* are common in both, those academics who do not tend to continue online education after Covid-19 put the emphasis on *education*, *interactivity*, and *efficiency*. The academics who tend to continue online education after Covid-19 speak of *reach* (massive number of learners), *location*, and *capacity*. The terminology associated with traditional face-to-face education in the classroom has been replaced in Figure 11 by a collection of concepts molded in favor of remote education, student-centered education, online courses, and open-access materials. Moreover, Figure 12 suggests that academics who do not forsake traditional teaching methods tend to characterize the word education as face-to-face education.

## Discussion and Conclusion

The global pandemic Covid-19 forced universities to offer online education, known as emergency remote teaching. However, the change on this scale was not a smooth transition, as many universities lacked the experience or the necessary infrastructure. The readiness to provide distance education and use Open Educational Resources (OERs) is threefold: at country, university, and individual levels. In Section *Emergency Remote Teaching: A Case Study on Higher Education in Turkey*, we primarily examined academics as individuals, how they have embraced this change, and whether they are willing to continue, and in Section *Investigation on Exploitation of OERs during Emergency Transition to Online Education* we additionally examine the efforts of the countries and universities in the sample.

Some higher education institutions had already established distance/hybrid education systems and were already producing/exploiting OERs. Some countries set up national YouTube channels and provide already available communication tools for general purposes to be used for distance education, such as e-mail. Some countries started working together to provide richer content to teachers and students in their region due to the pandemic. However, the sad fact is that there are countries whose education systems have already been disrupted by war and poverty that also maintain emergency distance education. To facilitate the process of emergency distance education while fighting Covid-19, some companies and institutions offer free access to OERs, some of which are shown in the timeline created (see Figure 1).

To deeply investigate to which extent academics use OERs preparing lectures during emergency remote teaching compared to their previous experience and knowledge on the subject was surveyed. The survey we conducted with academics from Turkey provides a deeper insight into academics’ thoughts about online learning and OERs in Section *Emergency Remote Teaching: A Case Study on Higher Education in Turkey* The findings show that while the majority of academics are suspicious of the effectiveness of online education as a substitute for face-to-face teaching, it is also found that many of them are not trained in preparing and delivering online lectures. One of the interesting results of our study is to show differences of opinion on OERs and distance learning between academics from different faculties and different roles. For example, academics from the arts, philology, and architecture are the most opposed to distance education, while academics from law, engineering, and health are the most positive (see Figure 9). Professors and lecturers are also the most opposed to distance education, while research assistants, who represent the youngest population in our data, are the most supportive of distance education (see Figure 10).

It is also shown that although there is not much difference between the female and male academics, the proportion of female academics who oppose distance education is greater than the proportion of male academics.

OERs as innovation in the context of our study was observed that the majority of academics do not know OERs and Creative Common (CC) licenses (Figure 7). In addition, our study confirms the findings of other studies (Koloğlu et al., 2016; Kurt, 2019) that academics are suspicious without having knowledge of OERs or have misconceptions (see Table 1). Of course, it is not reasonable to expect all academics to fully use OERs in their lectures as the last two stages of the adoption of innovation theory. However, we would like to give academics the opportunity to make their decision on the use of OERs on the basis of sufficient knowledge.

Regardless of their age, gender, academic role, and field of study, the majority of academics who participated in our study found the most difficult things during emergency remote teaching: online assessments, less engagement during lectures, less interaction, which is especially crucial in language learning, running tutorial courses, adapting lecture notes to the digital environment. Moreover, academics are confused about open licenses, how to create and share OERs and how to mix their lectures with OERs.

Our study found that many academics are not aware of the OERs that are freely available to them to design their digital learning content. Very few of them were familiar with OERs and open licenses and were able to use them effectively during emergency remote teaching. The misconceptions and reluctance of academics to use OER in their lectures and distance education in general (see sub-Section *Academics’ Future Plan on OERs and Online Learning* provide the arguments that academics in Turkey are mostly at the first stage of the theory of diffusion of innovations, namely Knowledge or Awareness Stage.

Our paper is proof that university lecturers do not know very well how to give sufficient online lectures and how to use OERs and licenses to keep up with changes in educational practice. Governments and open universities should lead national higher education policy on distance and blended learning teacher training. As Bozkurt (2020) suggested, we also believe that there must be support at the state level to create an open education culture and the necessary support and evaluation system to improve it.

We believe that our study paves the way for new studies. For example, there is a need to investigate the correlation and comparison of academics’ attitudes toward distance learning and OERs and the infrastructure provided by their institutions. Furthermore, one of the striking findings is that an academic, who suffers from bullying, finds distance learning and online teaching entirely satisfactory and mentally facilitating. Therefore, we have no evidence of what other participants experienced, but we certainly recommended that our fellow researchers conduct a study on bullying and home working during the pandemic.

As a good example of action, we can mention the European Commission’s Erasmus+ extraordinary 2020 call titled “Coronavirus response: Extraordinary Erasmus+ calls to support digital education readiness and creative skills” which encourages the applicants to use existing tools to enhance online, distance, and blended learning - including supporting teachers and trainers, as well as safeguarding the inclusive nature of digital learning opportunities (Erasmus+, 2020). The preliminary results of this study and other studies conducted across Europe during the pandemic inspired us to prepare a project to equip the academics with the necessary information and tools on digital pedagogy and, most importantly, provide the good practice examples and a collaborative platform to design a lecture face-to-face, online, or hybrid. The proposed project has been approved for funding [The name and the number of the project have been removed to keep the double-blind review process safe], and the project is in the stage of creating a digital hub for academics where they can find theory and practice together.

## Copyright statement

### Copyright

Copyright 2016© SAGE Publications Ltd, 1 Oliver’s Yard, 55 City Road, London, EC1Y 1SP, UK. All rights reserved.
